# Hot Aortic Nodules

**DOI:** 10.7759/cureus.10479

**Published:** 2020-09-16

**Authors:** Erik Soule, Quoc-Han Nguyen, Mario Dervishi, Jerry Matteo, Savas Ozdemir

**Affiliations:** 1 Interventional Radiology, University of Florida College of Medicine – Jacksonville, Jacksonville, USA; 2 Nuclear Medicine, University of Florida College of Medicine – Jacksonville, Jacksonville, USA

**Keywords:** atherosclerotic cardiovascular disease, inflammatory plaques, myocardial infarction, plaque rupture, cerebrovascular accident (stroke), fluorodeoxyglucose positron emission tomography

## Abstract

Atherosclerotic cardiovascular disease is the leading cause of death worldwide. Morbidity of the dreaded thrombotic complications of atherosclerosis such as cerebrovascular accident and myocardial infarction may be severe. Early detection of fulminant disease is therefore important for risk stratification and selecting a treatment strategy. In this report we present four patients in which 18-fluorodeoxyglucose uptake was identified in atherosclerotic plaques at positron emission tomography, performed for other indications. The study aims to showcase the potential implications of 18-fluorodeoxyglucose avid plaques, which may be otherwise overlooked at positron emission tomography. Early detection may aid in prevention of complications of atherosclerotic cardiovascular disease through aggressive lifestyle modification, as well as pharmacologic or other intervention, such as endovascular atherectomy.

## Introduction

The silent killer, atherosclerotic cardiovascular disease, is the leading cause of morbidity and mortality in the United States and globally. Atherosclerotic cardiovascular disease includes two widely known entities, ischemic heart disease (IHD) and cerebrovascular accident (CVA). Heart disease is the primary leading cause of death, while strokes are the third leading cause of death worldwide [1]. In 2010, the global cost of cardiovascular disease was estimated to be $863 billion. Between 2014 and 2015, the center for disease control estimated that IHD alone cost $219 billion in the United States. CVA is estimated to take 140,000 American lives, and cost $34 billion annually. Long-term disability is common with both IHD and CVA. Given the mortality and high level of morbidity, improvements in early detection, prevention, and treatment of atherosclerotic cardiovascular disease may be beneficial.

To understand atherosclerosis, it is important to look at the factors that increase its risks. A notable risk factor for atherosclerotic disease is inhaled tobacco use. The primary mechanism of atherosclerosis, however, is cholesterol formation. It is well documented that in adults with plasma cholesterol levels within the limits of normal, occurrence of symptomatic cardiovascular disease is very rare [2]. Beyond cholesterol formation, susceptibility of atherosclerosis is due to varying diameter arterial segments. The endothelium of these arterial segments undergoes shear forces and hemodynamic changes, predisposing to atherogenesis [2,3]. Under these conditions, the endothelium attracts atherogenic lipoproteins and a mosaic of inflammation begins. Monocytes begin to transmigrate the endothelium, subsequently differentiating into macrophages [2]. Macrophages play a vital role in plaque formation as they devour through the intima. As these macrophages undergo apoptosis, destabilized lipid-rich plaque forms. Furthermore, as additional macrophages arrive, they possess thrombogenic and destabilizing properties through their proteolytic enzymes [2].

The role of macrophages in the pathogenesis of atherosclerosis is key to the identification of potentially unstable plaques on positron emission tomography (PET) with computed tomography (CT). PET is a technique used in nuclear imaging to identify areas of interest through radiolabeled tracers [4-6]. Through a process called annihilation, these tracers give off photons and gamma rays. In PET-CT, radiolabeled glucose analogue 18-fluorodeoxyglucose (FDG) is utilized. Glucose is commonly taken up by metabolically active cells. In the case of atherogenesis, macrophages that are working hard in the intima increase expression of glucose transporters [4]. Lipid-laden macrophages may consequently accumulate in inflammatory atherosclerotic plaques. These ‘foamy’ macrophages have been hypothesized to represent a potential underlying cause of the phenomenon of radiolabeled glucose analogue 18-fluorodeoxyglucose uptake in atherosclerotic plaques, which has been postulated to indicate plaque instability [7].

## Case presentation

Case 1

**Figure 1:**
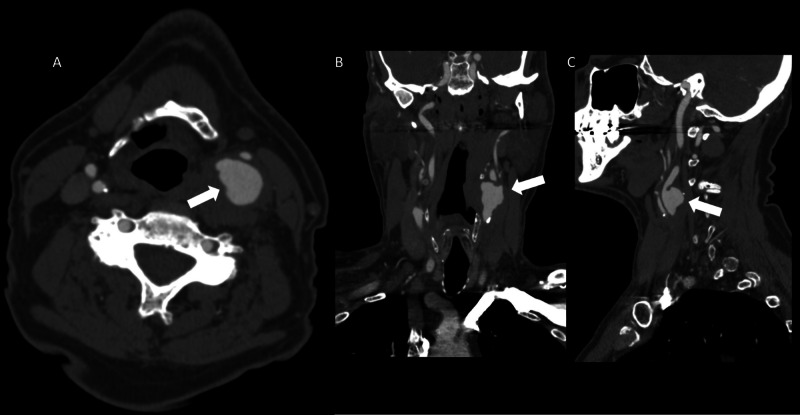
Axial (A), coronal (B), and sagittal (C) computed tomography images demonstrating a large pseudoaneurysm of the left internal carotid artery (white arrows).

A 70-year-old male with history of hypertension, hyperlipidemia, cerebrovascular accident, and 20 pack year smoking history presented to the emergency department with the chief complaint of hoarseness and one syncopal episode. Contrast enhanced computed tomography of the neck demonstrated a 3-cm left internal carotid artery (ICA) pseudoaneurysm (Figure 1A-1C).

The patient underwent open surgical resection of the carotid pseudoaneurysm with end to side anastomosis between the distal ICA and a large branch of the external carotid artery. The resected portion of the artery was sent for culture due to concern for superinfection. No evidence of fungal, bacterial, or mycobacterial involvement was identified. Histopathology was not performed. The patient returned to the hospital on post-operative day 15 due to wound dehiscence with purulent drainage. Surgical neck exploration was performed with copious irrigation utilizing an antibiotic solution. Wound cultures were positive for Cutibacterium acnes.

**Figure 2:**
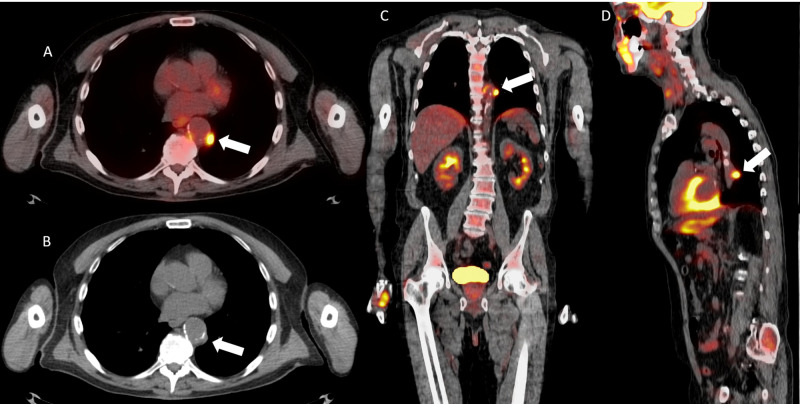
Axial positron emission tomography/computed tomography fusion image (A), axial computed tomography image (B), coronal (C), and sagittal (D) positron emission tomography/computed tomography fusion images demonstrating a fluorodeoxyglucose avid atherosclerotic plaque in the region of the descending aorta (white arrows).

Monoclonal gammopathy of unknown significance workup was underway at this time, and the patient underwent whole body PET/CT on postoperative day 40. No metabolically active neoplasm was identified, however, FDG uptake was seen within a nodular finding intimately associated with the wall of the proximal descending aorta (Figure 2A-2D).

**Figure 3:**
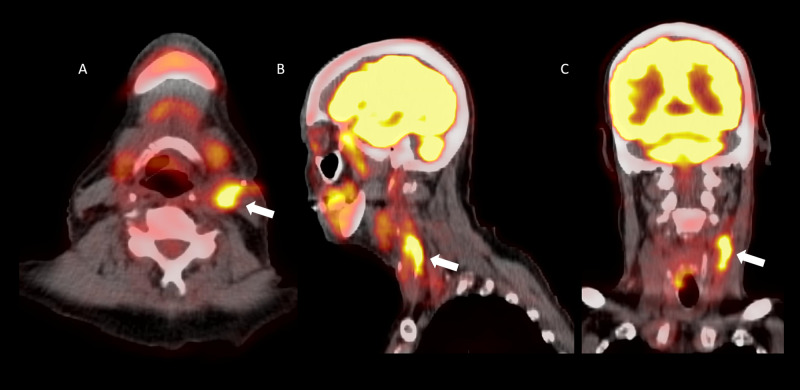
Axial (A), sagittal (B), and coronal (C) positron emission tomography/computed tomography fusion images demonstrating fluorodeoxyglucose uptake in the region of the operative bed (white arrows).

Additional FDG uptake was seen in the region of the operative bed, which was attributed to postoperative inflammation (Figure 3A-3C).

The PET avid atherosclerotic plaque within the aorta is thought to be related to fulminant atherosclerotic disease due to numerous risk factor including an extensive smoking history. This may additionally represent the underlying pathophysiology of the carotid pseudoaneurysm.

Case 2

**Figure 4:**
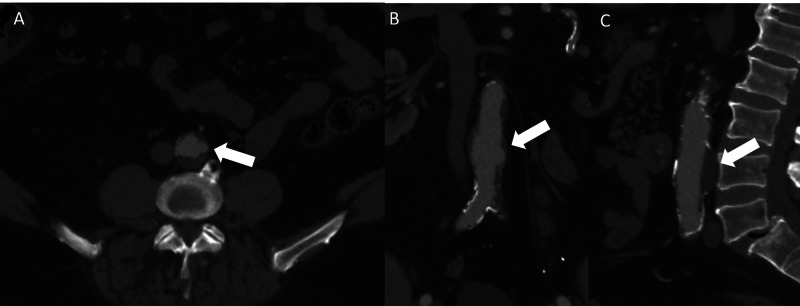
Axial (A), coronal (B), and sagittal (C) computed tomography images demonstrate a partially thrombosed abdominal aortic aneurysm (white arrows).

A 74-year-old male patient with past medical history of obesity, hypertension, coronary artery disease status post coronary artery bypass grafting, and cerebrovascular accident, with a remote 40 pack year smoking history presented with abdominal pain. He underwent computed tomography angiography of the chest, abdomen and pelvis which demonstrated an unruptured 3 cm abdominal aortic aneurysm (Figure 4A-4C) as well as an incidental dilated appendix.

**Figure 5:**
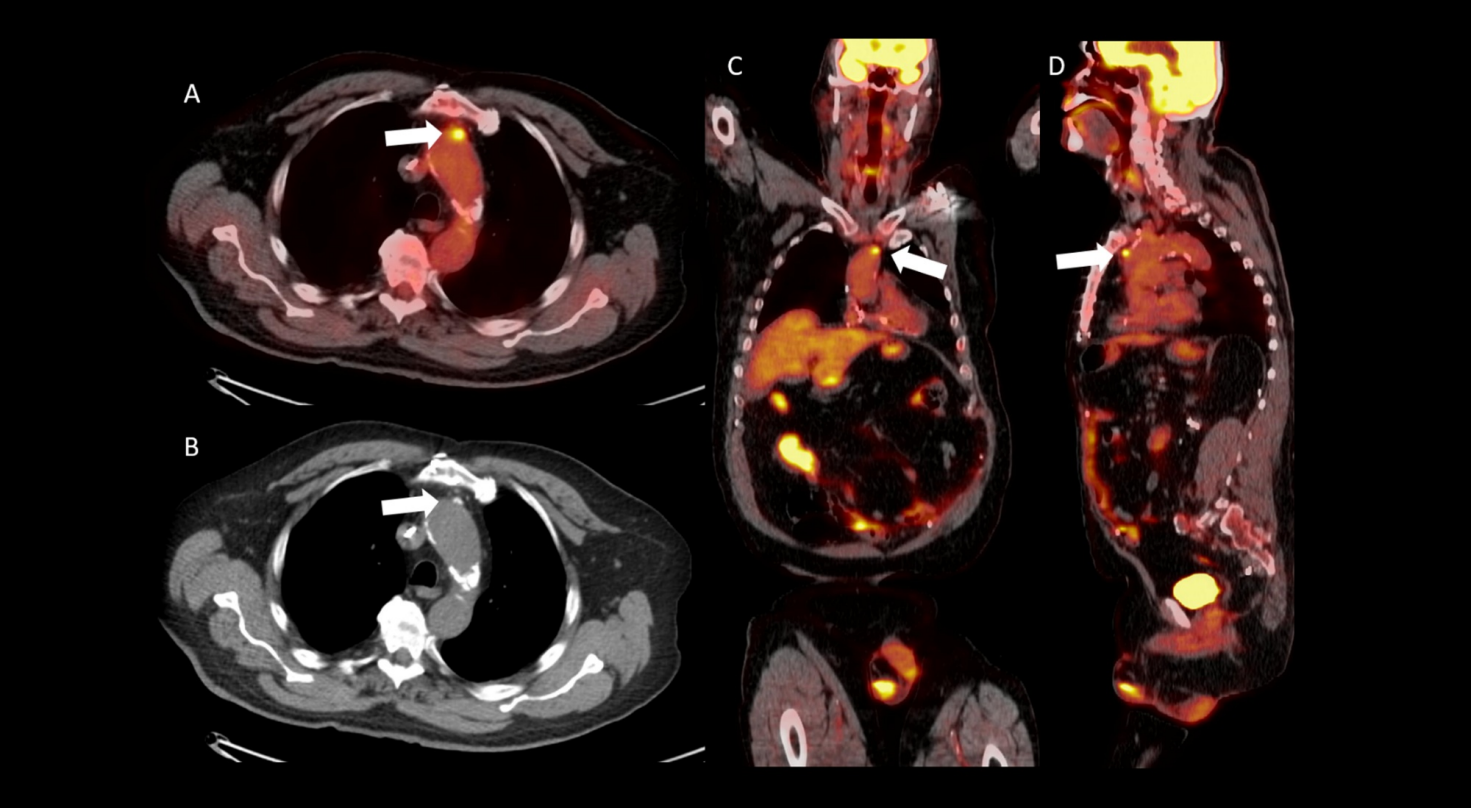
Axial positron emission tomography/computed tomography fusion image (A), axial computed tomography image (B), coronal (C), and sagittal (D) positron emission tomography/computed tomography fusion images demonstrating a fluorodeoxyglucose avid atherosclerotic plaque in the region of the aortic root (white arrows).

The appendix was resected, and the pathologic specimen demonstrated mucinous adenocarcinoma of the appendix. Workup for metastatic disease including PET/CT demonstrated several nodular, FDG-avid findings at the aortic root (Figure 5A-5D).

**Figure 6:**
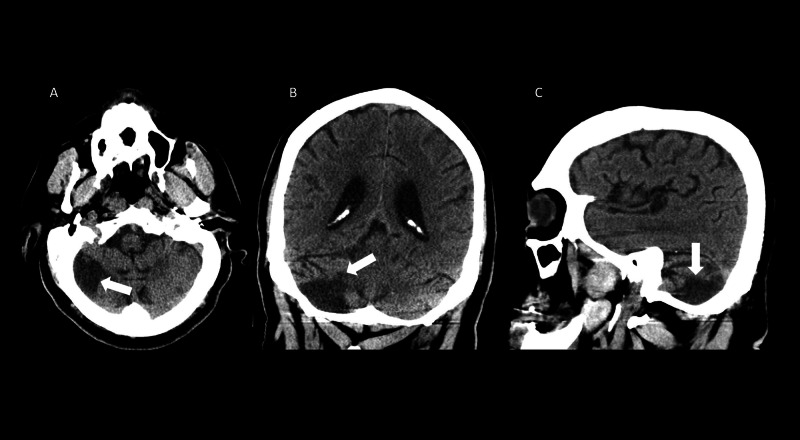
Axial (A), coronal (B), and sagittal (C) computed tomography images demonstrating encephalomalacia in the right cerebellar hemisphere consistent with prior infarct (white arrows).

The abdominal aortic aneurysm was not FDG avid although review of prior imaging demonstrated complications of atherosclerotic disease including a right posterior inferior cerebellar artery distribution infarct (Figure 6A-6C).

At follow-up PET/CT, the FDG avid aortic lesions had resolved. The abdominal aortic aneurysm has remained stable.

Case 3

**Figure 7:**
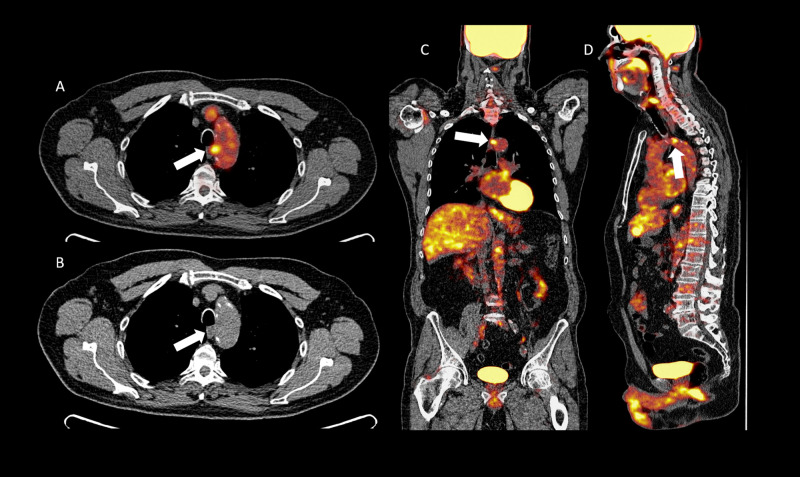
Axial positron emission tomography/computed tomography fusion image (A), axial computed tomography image (B), coronal (C), and sagittal (D) positron emission tomography/computed tomography fusion images demonstrating a fluorodeoxyglucose avid atherosclerotic plaque in the region of the aortic arch (white arrows).

A 69-year-old male with past medical history of hypertension, obesity, and a history of cigar smoking presented with a pulmonary nodule. He received a PET/CT which demonstrated an FDG-avid finding intimately associated with the aortic arch (Figure 7A-7D).

The pulmonary nodule was thought to represent a calcified granuloma. The patient has not received a follow-up PET/CT, and to date has not presented at this institution with a thrombosis event.

Case 4

**Figure 8:**
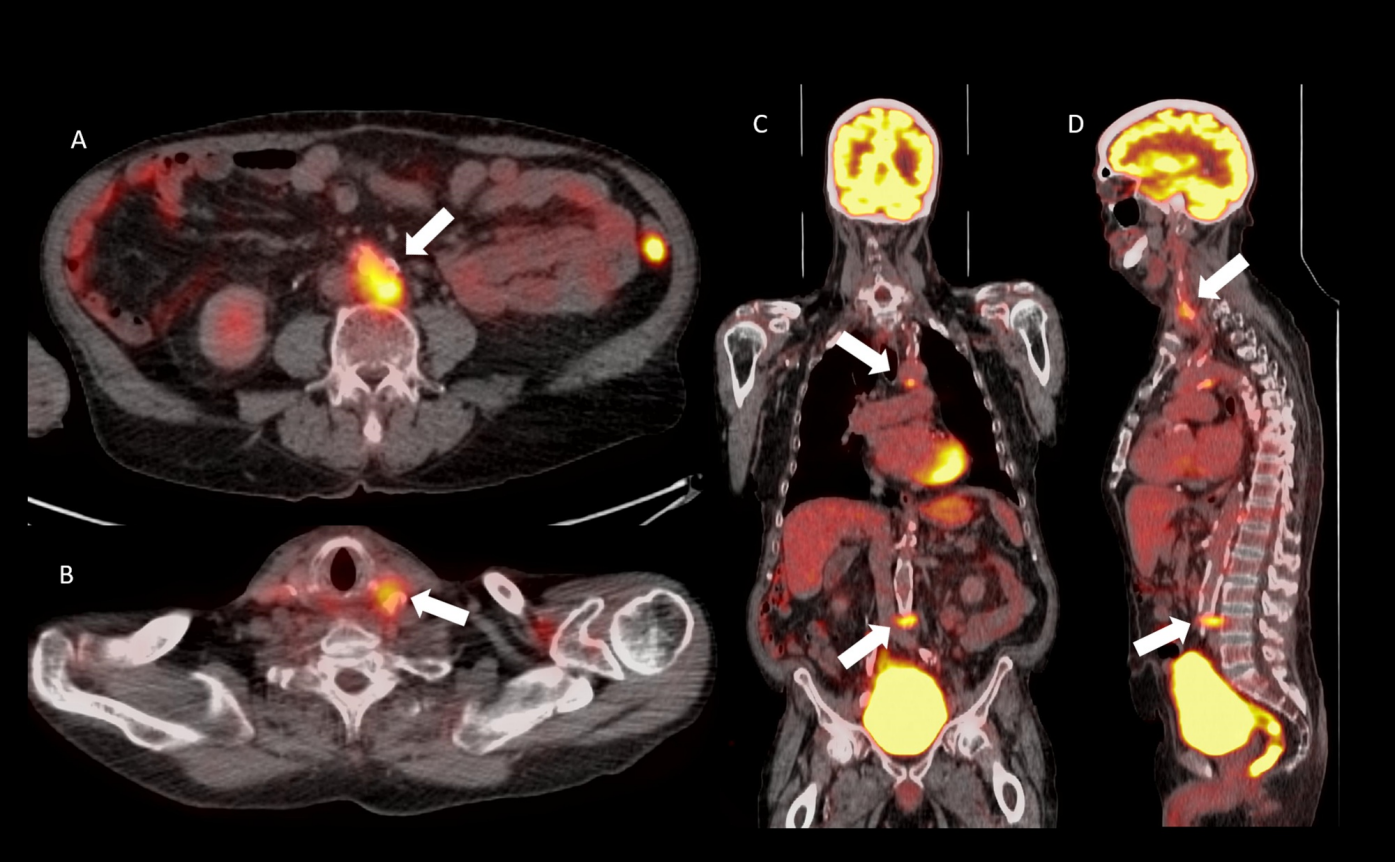
Axial positron emission tomography/computed tomography fusion image (A), axial computed tomography image (B), coronal (C), and sagittal (D) positron emission tomography/computed tomography fusion images demonstrating fluorodeoxyglucose avid atherosclerotic plaques in the regions of the aortic bifurcation, aortic arch, and left carotid (white arrows).

A 71-year-old male with coronary artery disease, congestive heart failure, type II diabetes mellitus, hypertension and a history of cigar smoking presented with hoarseness. He was subsequently found to have supraglottic squamous cell carcinoma of the larynx. During workup for metastatic disease, a PET/CT was performed, demonstrating FDG-avid atherosclerotic lesions involving the aortic arch, aortic bifurcation, and left carotid (Figure 8).

He subsequently underwent chemoradiation and ceased tobacco use. Upon follow-up PET/CT, FDG uptake in the atherosclerotic lesions was abrogated.

## Discussion

These four cases demonstrate the potential of FDG-PET to localize arterial areas of concern in patients with risk factors for atherosclerotic disease [8]. With follow-up, several FDG-avid lesions were abrogated in patients who achieved smoking cessation. Beyond our four case studies, similar findings were observed in meta-analysis by Chowdhury et al. showing a difference in radionucleotide uptake in symptomatic versus asymptomatic carotid disease [4]. In the abdominal aorta, Higashigawa et al. demonstrated that dissected aortic walls had higher levels of FDG uptake [9].

The potential to identify metabolic activity in atherosclerotic plaques with FDG-PET allows for functional assessment of atherosclerotic disease beyond the traditional paradigm of stenosis measurements to guide treatment. Additional imaging studies may be considered to further characterize FDG-avid plaques such as ultrasound or dedicated MRI [10]. Treatment for atherosclerotic disease ranges from lifestyle modification and pharmacologic treatment to more invasive interventions such as angioplasty, stenting, atherectomy, and open surgery. It may be preferable to intervene in high-risk patients before a devastating thrombosis event such as CVA or myocardial infarction occurs. Therefore, there may be a role for FDG-PET in risk stratification to intervene in a timely fashion and thus decrease the incidence of catastrophic thrombosis events in high-risk patients [11].

Other potential uses for FDG-PET include surveillance of pharmacologic efficacy in abrogating FDG avidity of atherosclerotic plaques [12]. This may allow for individualized treatment with a customized regimen. For example, a patient with continued evidence of FDG-avid plaques despite medical therapy may be prescribed additional medications, additional lifestyle modifications, or a different therapeutic approach could be taken, such as invasive therapy. This may allow for a patient centered approach utilizing physiologic information regarding metabolic activity of atherosclerotic plaques gained from FDG-PET.

## Conclusions

Even with advancements in treatment of atherosclerotic cardiovascular disease, it continues to be the leading cause of death in the United States and abroad. PET-CT scan may provide valuable functional information about atherosclerotic plaques in cardiovascular disease, even in patients undergoing traditional screening and management. PET-CT has the potential to play a role in early detection of high-risk atherosclerotic disease. This modality may allow an individualized approach to cardiovascular disease, potentially reducing the incidence of catastrophic thrombotic events such as cerebral and myocardial ischemia/infarction.
